# Inpatient care burden due to cancers in Anhui, China: a cross-sectional household survey

**DOI:** 10.1186/s12889-016-2995-z

**Published:** 2016-04-11

**Authors:** Ting Zhao, Jing Cheng, Jing Chai, Rui Feng, Han Liang, Xingrong Shen, Rui Sha, Debin Wang

**Affiliations:** School of Health Services Management, Anhui Medical University, 81 Meishan Road, Hefei, 230032 Anhui China; Department of Epidemiology and Statistics, School of Public Health, Anhui Medical University, 81 Meishan Road, Hefei, 230032 China; Library Department of Literature Retrieval and Analysis, Anhui Medical University, 81 Meishan Road, Hefei, China

**Keywords:** Cancer, Costs, Inpatient care, Household survey, Financial burden

## Abstract

**Background:**

The financial burden of cancers has profound effects and there is a clear need to explore the issue from different perspectives and for different population groups. This study aimed at investigating inpatient cancer care (ICC) burden in Anhui, a typical inland province of China.

**Methods:**

The study collected data through a household survey conducted during April to November, 2014 using cluster-randomized sampling and a structured questionnaire administered face-to-face by trained interviewers.

**Results:**

The survey covered 60,678 urban and rural residents and 318 person-times of ICC during the past year. Age-adjusted annual person-times and days of ICC per thousand population added up to 4.24 and 76.78 respectively and urban residents showed significantly greater admission rates and length of stay than that of rural ones. Total ICC expenditures accounted for 13.30 % of all that of inpatient care for the whole population. Per-case direct and indirect costs of all types of cancers were estimated as 10365.1 and 929.9 RMB. Per-case total cost on ICC at township hospitals was 2142.3 RMB and at province level hospitals, 17133.0 RMB. Significant variations in per-case ICC expenditures also existed between patients with different household income and type of medical insurance systems, but patients suffering from different types of cancers. Out-of-pocket payment due to ICC turned out to be catastrophic for 20.6 % of all cancer patients and 65.2 % for other medical insurance, 45.6 % for enrollees of urban and rural medical insurance, 43.2 % for the 65 to 74 years old. Multi regression revealed statistically significant association between ICC costs and education, reimbursement ratio, household income and level of hospital.

**Conclusions:**

Cancers characterize low incidence, moderate service use and high expenses. There exist substantial differences between subgroups and part of these variations cannot be explained by pathological factors. ICC expenses are catastrophic in nature to a large part of patients. There is a clear need for more effectively regulating cancer-related medical practices and service seeking behaviors.

**Electronic supplementary material:**

The online version of this article (doi:10.1186/s12889-016-2995-z) contains supplementary material, which is available to authorized users.

## Background

Cancers incur tremendous financial burden on patients, their relatives and health care systems. Estimated annual total cost of cancers added up from $1643 to $7705 per patient [1–4]. Estimated per-case cancer expenditure ranged from a few percent to several times of the family income of the affected [5]. Cancers incur financial burdens through several ways. These include direct input on the diagnosis and treatment of the diseases, indirect costs spent on travel to and from service providers, supplementary nutrients and nursing care etc., and income loss due to disease-related absenteeism or retirement, unemployment, bankruptcies and discriminations [1, 4, 6–13]. Cancer expenditures have witnessed rapid increase during the past decades [14, 15]. And this fast growing trend is most likely to continue in the future. On the one hand, aging population and deteriorating environments and lifestyles are causing an increasing cancer epidemiology [16]. On the other, people diagnosed with cancers survive longer and longer due to improving early detection technologies, expanding screening services, and emerging new therapies [16, 17]. Besides, these are paralleled by surging overall and healthcare consumption in general and relatively loose cost-containment and clinical guidelines on cancer services in particular.

Perhaps, the first and hardest hit by financial burden of cancers (FBC) may be the patients and their relatives, for whom it has profound effects and implications by various means. First, FBC affects the outcomes of cancer case management. Zafar and colleagues conducted a cohort study of 1000 colorectal or lung cancer patients, and found that financial burden was closely related to the patients’ health-related quality of life [18]. The study by Zarogoulidou et al. discovered significant relationships between direct cost on chemotherapy and overall survival, quality of life and treatment outcome [19]. Second, FBC influences cancer treatment decision-making and compliance. Streeter and coworkers reported that patients with cost sharing greater than $500 were 4.46 times more likely to abandon oral oncolytic treatment than patients with cost sharing of $100 or less [20]. Similarly, Zafar et al. found that, when faced with financial difficulties, cancer patients tend to take less than the prescribed amount of medication (20 %), partially fill prescriptions (19 %) or avoid filling prescriptions altogether (24 %) [21]. Third, FBC aggravates cancer induced negative worries and feelings. A cross-sectional survey of cancer patients seeking financial assistance from the HealthWell Foundation revealed that higher financial burden was also associated with dissatisfaction with the technical quality of health care [22].

Given the magnitude and influences of FBC, there is a clear need to explore the issue from different perspectives and for different population groups. This especially applies to China, a nation with the largest population and number of cancer cases and the least insurance schemes for cancer care. Most pharmaceutical regimens and surgical and radiological therapies for cancer patients in China fall out of the government’s list of essential medicine and are hence ineligible for any kinds of government subsidy. Compared with the emerging literature documenting FBC and its influences in other countries (mostly the developed nations), there is a general paucity in publications on the same issue in China. This study aimed at investigating inpatient cancer care (ICC) burden in Anhui, a typical inland province of China, using data derived from a household survey.

## Methods

### Data collection

The study used part of the data obtained through a household survey conducted during April to November, 2014 in Anhui, a typical inland province of China. The survey aimed at assessing health services need and utilization by urban and rural residents and informing health resources planning. It adopted a structured questionnaire administered face-to-face at the respondents’ households by trained interviewers. Variables surveyed comprised two main aspects, i.e., socio-demographics and healthcare utilization and costs. The former consisted of age, gender, education, household income, type of residence (urban vs. rural), and insurance(s) enrolled. The latter solicited information about all episodes of health services use happened during the past 12 months by all the household members including type of service (i.e., outpatient or inpatient care) received, name and classification code of the disease which caused the service use, self-reported direct and indirect costs incurred due to the service, and level of the service provider. Selection of the households employed a stratified random sampling proceeded in two stages. Stage 1 classified all the counties and cities in Anhui province into southern, northern and middle areas. Stage 2 randomly selected: a) 6 counties and 6 cities from each of the 3 areas (36 counties/cities in total); b) 5 communities from each of the counties or cities; c) 120 household from each of the communities.

### Data analysis

The study conducted mainly descriptive analysis using SPSS version 16. It calculated, for different groups, the average values of: a) annual person-times and person-days of ICC; b) per case ICC expenditures; c) percentages of ICC expenditure versus all diseases; and d) ICC expenditure reaching given cutoff percentages of family income. It also performed multivariate regression analysis and power tests to detect statistical differences using *χ*^2^ test, or rank sum test or ANOVA with α < 0.05 being considered significant. In order to control age-induced confounding, the study groups were standardized using the national average age distributions in 2010 generated from the latest China population census. Given that the direct and indirect health expenditures displayed severely skewed distributions, they were translated into normal distributions to enable statistical power tests using Ln(Ln(x)), where x stands for individual health expenditures. To facilitate international comparison, the inpatient care expenditures in Chinese yuan (RMB) were translated into US dollars ($) at $1 = 6.2 RMB, the average exchange rate for the year of 2013 when the survey was carried out. Considering that researchers hold different views about how to define catastrophic health expenditures [23], the study calculated catastrophic ICC expenditures using a series of cutoff values, i.e., 20, 30, 40 and 50 % of total household income.

### Ethics, consent and permissions

The study protocol had been reviewed and approved by the Biomedical Ethics Committee of Anhui Medical University. Participation of subjects was voluntary and written consent was obtained from all participants prior to data collection.

## Results

### Characteristics of subjects surveyed and ICC cases

The household survey reached response rate of 86.68 % and obtained data about 60,678 residents consisting of 29,980 males and 30,698 females and 30,597 urban and 30,081 rural residents (Table 1). They reported a total of 318 person-times of ICC during the past year including 178 and 140 person-times for male and female patients and 171 and 147 person-times for urban and rural patients respectively.

**Table 1:** Descriptive statistics of standardized inpatient cancer care cases and subjects surveyed

Grouping criteria	Hospital admissions (person-times)					Subjects surveyed (×1000)				
	Total	Males	Females	Urban	Rural	Total	Males	Females	Urban	Rural
Age (years)										
Under 45	70	14	56	48	22	31.05	15.42	15.63	16.08	14.96
45 to 54	62	29	33	37	25	10.45	4.88	5.57	5.40	5.06
55 to 64	100	67	33	42	58	9.81	4.90	4.91	4.64	5.18
65 to 74	44	33	11	22	22	6.06	3.19	2.86	2.92	3.14
75 or over	42	35	7	22	20	3.31	1.58	1.73	1.57	1.74
Type of health insurance										
NCMC	126	73	51	35	91	35.69	17.32	18.37	12.77	22.92
CEMI	42	18	22	41	0	6.82	3.87	2.95	6.34	0.48
CRMI	39	9	34	37	1	6.26	2.78	3.48	5.66	0.60
URMI	13	9	4	2	11	4.92	2.40	2.52	1.71	3.21
Others	16	9	7	11	4	6.98	3.61	3.37	4.12	2.86
Household income (quartile)										
Q1	53	27	26	18	33	10.75	5.32	5.43	4.68	6.07
Q2	70	23	49	48	22	19.19	9.39	9.80	8.92	10.27
Q3	78	45	32	42	36	16.80	8.28	8.52	9.08	7.73
Q4	72	37	33	48	26	13.88	6.96	6.92	7.88	6.01
Education										
No education	41	19	22	22	20	17.51	6.53	10.97	7.28	10.23
Primary school	73	39	26	23	49	16.55	8.30	8.25	7.28	9.27
Middle school	71	41	20	35	38	16.62	9.40	7.22	8.69	7.93
Secondary or higher school	58	21	33	55	6	10.00	5.75	4.25	7.35	2.65
Total	257	121	135	146	111	60.68	29.98	30.70	30.60	30.08

Note: *NCMC* new corporative medical care, *CEMI* city employee medical insurance, *CRMI* city resident medical insurance, *URMI* urban and rural medical insurance

### Person-times and person-days of ICC per thousand population

Table 2 provides standardized figures about person-times and person-days of ICC in the past year by different groups. On average, 1000 people studied incurred 4.24 person-times of hospital admissions and 76.78 person-days of ICC in the past 12 months and urban residents presented significantly greater admission rates and length of stay (days) than that of rural respondents. The two indicators showed substantial discrepancies between different subgroups varying from 0.00 person-times (for rural enrollees of city employee medical insurance) to 22.11 person-times (for males aged 75 years or over) and from 13.03 person-days (for residents with secondary or higher education in rural areas) to 382.28 person-days (for males aged 75 years or over) respectively. The majority (83 out of 116) of these differences were statistically significant with clear trends being observable within age subgroups of males, females, urban residents, and rural residents.

**Table 2:** Standardized annual person-times and person-days of inpatient cancer care per thousand populations

Grouping criteria	Person-times of hospital admissions							Person-days of hospital stay						
	Total	Males (M)	Females (F)	P (M vs. F)	Urban	Rural	P (U vs. R)	Total	Males	Females	P (M vs F)	Urban	Rural	P (U vs R)
Age (years)														
Under 45	2.25	0.91	3.58	<0.001	2.98	1.47	0.005	34.05	23.35	44.61	<0.001	31.63	36.63	<0.001
45 to 54	5.93	5.94	5.93	0.994	6.86	4.94	0.202	126.40	143.10	111.70	<0.001	168.64	81.26	<0.001
55 to 64	10.19	13.67	6.72	0.001	9.06	11.20	0.291	180.98	233.13	128.91	<0.001	191.34	171.75	<0.001
65 to 74	7.26	10.34	3.84	0.003	7.54	7.00	0.805	158.80	219.21	91.49	<0.001	157.08	160.46	0.405
75 or over	12.69	22.11	4.06	<0.001	14.05	11.47	0.509	233.54	382.28	97.33	<0.001	275.21	196.21	<0.001
*P* (value)	<0.001	<0.001	0.026	NA	<0.001	<0.001	NA	<0.001	<0.001	<0.001	NA	<0.001	<0.001	NA
Type of health insurance														
NCMC	3.53	4.22	2.78	0.021	2.74	3.97	0.061	54.87	63.05	45.72	<0.001	43.51	60.90	0.032
CEMI	6.16	4.65	7.46	0.131	6.47	0.00	0.076	145.62	152.11	135.65	<0.001	154.34	34.08	<0.001
CRMI	6.23	3.24	9.76	0.002	6.54	1.66	0.142	74.68	83.85	80.73	<0.001	80.74	16.05	<0.001
URMI	2.64	3.75	1.59	0.139	1.17	3.43	0.142	86.95	137.16	45.82	<0.001	32.30	116.34	<0.001
Others	2.29	2.49	2.07	0.714	2.67	1.40	0.259	61.25	56.23	61.65	<0.001	56.18	61.44	<0.001
*P* (value)	<0.001	0.537	<0.001	NA	<0.001	0.125	NA	<0.001	<0.001	<0.001	NA	<0.001	<0.001	NA
Household income (quartile)														
Q1	4.93	5.08	4.79	0.828	3.85	5.44	0.235	63.51	66.98	58.44	<0.001	47.75	73.02	<0.001
Q2	3.65	2.45	5.00	0.004	5.38	2.14	<0.001	53.17	48.67	58.08	0.051	72.82	35.64	<0.001
Q3	4.64	5.43	3.76	0.116	4.63	4.66	0.769	73.91	85.42	61.15	<0.001	69.73	78.88	0.083
Q4	5.19	5.32	4.77	0.603	6.10	4.33	0.092	144.32	160.27	129.58	<0.001	157.88	133.54	<0.001
*P* (value)	<0.001	<0.001	0.021	NA	0.008	<0.001	NA	<0.001	<0.001	<0.001	NA	<0.001	<0.001	NA
Education														
No education	2.34	2.91	2.01	0.232	3.02	1.96	0.155	47.00	45.47	44.70	0.378	50.09	45.28	0.052
Primary school	4.41	4.70	3.15	0.112	3.16	5.28	0.039	74.65	76.13	62.51	<0.001	52.94	91.74	<0.001
Middle school	4.27	4.36	2.77	0.092	4.03	4.79	0.457	86.66	100.52	39.69	<0.001	88.96	81.11	<0.001
Secondary or higher school	5.80	3.65	7.76	0.006	7.48	2.26	0.003	108.23	93.19	119.02	<0.001	140.40	13.03	<0.001
*P* (value)	<0.001	0.327	<0.001	NA	<0.001	<0.001	NA	<0.001	<0.001	<0.001	NA	<0.001	<0.001	NA
Total	4.24	4.04	4.40	0.492	4.77	3.69	0.040	76.78	82.97	70.50	<0.001	82.52	71.97	<0.001

Note: *NCMC* new corporative medical care, *CEMI* city employee medical insurance, *CRMI* city resident medical insurance, *URMI* urban and rural medical insurance, the power for the differences in person-times of hospital admissions among different groups was calculated using *χ* ^2^ test and that in person-days of hospital stay, rank sum test

### ICC expenditures by cancer types and population groups

**Fig. 1:**
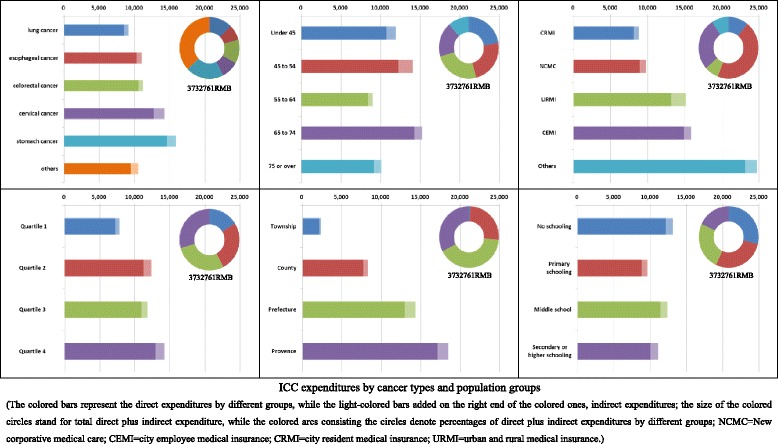
ICC expenditures by cancer types and population groups. The *colored bars* represent the direct expenditures by different groups, while the *light-colored bars* added on the right end of the colored ones, indirect expenditures; the size of the *colored circles* stand for total direct plus indirect expenditure, while the *colored arcs* consisting the circles denote percentages of direct plus indirect expenditures by different groups; NCMC = New corporative medical care; CEMI = city employee medical insurance; CRMI = city resident medical insurance; URMI = urban and rural medical insurance

Figure 1 and Appendix 1 and 2 show average and percentages of ICC expenditures by different cancer types and population groups. Putting together, per-case direct and indirect costs of all types of cancers were estimated as 10365.1 RMB (or $1671.8) and 929.9 RMB ($150.0) respectively. Stomach cancer incurred the highest direct expenditure (14627.7 RMB or $2359.3) followed by cervical cancer (12741.5 RMB or $2055.1), liver cancer (10909.0 RMB or $1759.5) and colorectal cancer (10584.8 RMB or $1707.2); while liver cancer, the highest indirect cost (1535.4 RMB or $247.6) followed by cervical cancer (1520.9 RMB or $245.3), nasopharyngeal cancer (1344.5 RMB or $216.9), and breast cancer (1326.3 RMB or $213.9). Most of the mean expenditures turned out to be smaller than the corresponding standard deviations (SD) and all mean expenditure, greater than the corresponding median expenditure. Per-case ICC expenditures by different groups of patient characteristics also witnessed similar features, e.g., large variations across subgroups, greater standard deviations than means and means than medians. For the 5 categories of subgroups (e.g., age, household income) listed, 4 of the intra-group differences in direct and indirect per-case ICC expenditures were statistically significant (*p* < 0.05). Some extent of increasing trends (i.e., higher grade of the grouping variable, higher indirect and indirect expenditure) were observable among subgroups of level of hospital, household income, and age. All the 318 cancer ICC caused a total of 3,732,761 ($602058.2) RMB direct (indirect) costs, with stomach cancers incurred the most (20.36 %) among all cancer types followed by colorectal cancer (12.54 %); and the 55 to 64 years (24.34 %) among all age groups, and prefecture hospitals (40.95 %) among all levels.

### Expenditures on inpatient care for cancers vs. all diseases

The 60,678 residents reported 5026 person-times of inpatient care during the past 12 months. Of these, ICC episodes accounted for 6.33 %. Table 3 presents the standardized percentages of inpatient care expenditures due to cancers versus all diseases for all the study population under concern. Looking at overall data, the direct and indirect ICC expenditures accounted for 13.30 and 12.70 % of the inpatient care expenditures for the whole population studied (*N* = 60,678). Turning to specific figures for different subgroups, the percentages ranged from 2.53 to 25.23 % (for direct ICC expenditure) or 2.20 to 30.55 % (for indirect ICC expenditure) with the highest percentage of direct expenditures being found with urban residents with secondary or higher education (25.23 %) followed by rural enrollees of urban and rural medical insurance (25.21 %), urban patients aged from 45 to 54 years old (24.51 %), female enrollees of city employee medical insurance (23.48 %), females with second high quartile of household income (23.38 %).

**Table 3:** Standardized percentage of inpatient care expenditure due to cancers versus all diseases

Grouping criteria	Direct expenditure (%)					Indirect expenditure (%)				
	Total	Males	Females	Urban	Rural	Total	Males	Females	Urban	Rural
Age (years)										
Under 45	13.91	12.98	14.83	15.10	12.76	17.19	17.38	17.06	18.15	16.32
45 to 54	21.82	21.02	22.55	24.51	18.60	22.90	28.22	17.99	30.55	15.16
55 to 64	18.56	22.05	14.46	19.43	17.55	12.13	11.37	13.14	12.23	12.06
65 to 74	11.72	16.89	6.26	8.91	16.36	11.94	16.51	6.57	5.99	19.07
75 or over	16.84	23.23	7.65	14.75	21.72	15.40	19.27	8.92	13.83	17.19
Type of health insurance										
NCMC	9.42	9.07	8.12	7.36	10.35	8.70	8.49	7.99	7.10	9.41
CEMI	19.75	16.87	23.48	20.31	8.48	21.45	20.61	24.90	22.52	1.80
CRMI	17.01	20.88	16.26	16.99	13.60	12.77	11.46	14.89	12.82	10.96
URMI	17.76	14.41	9.69	4.36	25.21	13.53	15.45	8.67	6.43	18.59
Others	17.03	14.23	18.21	20.10	10.14	14.05	12.41	15.09	11.39	18.99
Household income (quartile)										
Q1	8.17	2.53	12.84	7.28	8.70	6.49	2.20	9.03	5.44	7.19
Q2	16.89	10.29	23.38	21.81	12.06	18.30	9.86	25.33	26.06	10.63
Q3	12.91	11.98	10.52	13.18	12.41	8.89	9.68	7.20	5.30	12.46
Q4	16.73	16.51	14.44	17.78	16.44	16.44	16.62	15.63	17.27	15.74
Education										
No education	11.78	10.58	13.07	12.36	12.48	8.35	9.01	9.24	8.46	9.54
Primary school	13.47	11.79	12.03	10.66	17.28	15.38	12.45	18.91	11.15	20.31
Middle school	13.48	13.06	9.56	13.32	13.52	7.78	8.30	4.53	6.21	9.55
Secondary or higher school	19.76	15.41	7.63	25.23	3.23	24.21	24.77	21.82	30.18	3.77
Total	13.30	11.11	13.34	14.18	12.49	12.70	10.97	13.56	13.41	11.99

Note: *NCMC* new corporative medical care, *CEMI* city employee medical insurance, *CRMI* city resident medical insurance, *URMI* urban and rural medical insurance

### Catastrophic features of ICC expenditures

Table 4 compares the standardized percentages of un-reimbursed ICC expenditures (or expenditures paid by patients rather than governments or insurance companies) that had reached 20, 30, 40 and 50 % of household income respectively. Judging by the World Health Organization standard (40 % of household income) [24], as high as 20.6 % of the single episode ICC expenditure turned out to be catastrophic to the patients. The chances of catastrophic ICC expenditure witnessed substantial differences among different subgroups and ICC expenditure was more likely to be catastrophic for patients who were males, rural residents, and enrollees of urban and rural medical insurance.

**Table 4:** Standardized percentage of inpatient cancer care expenditure reaching given cutoff percentages of family income

Grouping criteria	Direct expenditure meeting given % income				Total expenditure meeting given % income			
	20 %	30 %	40 %	50 %	20 %	30 %	40 %	50 %
Age (years)								
Under 45	21.4	17.1	14.3	12.9	22.9	20.0	20.0	15.7
45 to 54	41.9	32.3	27.4	22.6	51.6	41.9	33.9	27.4
55 to 64	30.0	22.0	18.0	14.0	36.0	26.0	24.0	18.0
65 to 74	56.8	43.2	43.2	38.6	59.1	50.0	43.2	43.2
75 or over	35.7	19.0	14.3	14.3	40.5	21.4	19.0	14.3
*P* value	0.002	0.012	0.002	0.004	0.001	0.002	0.034	0.003
Gender								
Males	26.6	18.4	16.4	14.0	30.0	21.6	20.1	16.9
Females	24.3	19.7	15.6	13.6	29.3	24.4	20.7	16.1
*P* value	0.062	0.490	0.298	0.486	0.103	0.658	0.314	0.240
Region								
Urban	22.0	16.1	14.0	12.8	26.0	19.9	17.0	14.5
Rural	31.0	23.1	19.1	15.5	35.2	27.5	25.4	19.7
*P* value	0.070	0.091	0.126	0.348	0.075	0.161	0.082	0.188
Type of health insurance								
NCMC	27.8	18.8	15.4	12.4	31.7	23.8	21.1	16.3
CEMI	9.2	8.6	7.6	7.6	12.8	9.2	8.6	8.6
CRMI	20.5	14.8	11.4	9.1	24.3	16.8	14.8	9.1
URMI	50.1	41.9	38.6	36.0	50.1	45.6	45.6	38.6
Others	81.1	65.2	57.2	46.8	88.3	80.4	65.2	57.2
*P* value	<0.001	<0.001	<0.001	0.001	<0.001	<0.001	<0.001	<0.001
Household income (quartile)								
Q1	20.4	14.9	12.9	12.5	22.5	17.0	16.6	12.9
Q2	23.0	20.5	18.1	15.7	32.4	24.0	19.6	17.1
Q3	41.7	26.8	21.0	15.7	44.5	33.1	31.1	22.5
Q4	21.5	14.8	12.6	11.4	23.7	17.3	13.7	13.7
*P* value	0.008	0.091	0.103	0.123	0.005	0.031	0.012	0.153
Level of hospital								
Township	15.4	7.7	7.7	7.7	23.1	7.7	7.7	7.7
County	31.1	20.5	17.2	14.8	37.7	24.6	21.3	17.2
Prefecture	43.4	31.9	26.5	23.9	46.9	39.8	34.5	29.2
Provence	31.4	27.1	25.7	20.0	35.7	30.0	28.6	22.9
*P* value	0.076	0.098	0.164	0.228	0.222	0.025	0.062	0.104
Education								
No education	26.8	21.5	19.6	19.1	32.7	25.8	23.8	20.2
Primary school	21.2	15.5	14.0	11.2	23.0	18.5	16.2	13.3
Middle school	28.6	17.5	11.2	7.6	35.5	21.9	16.7	12.4
Secondary or higher school	19.4	16.0	14.5	14.5	21.0	17.9	16.0	16.0
*P* value	0.001	0.010	0.006	0.001	<0.001	0.003	0.003	0.019
Total	25.9	19.1	16.3	14.0	30.0	23.1	20.6	16.7

Note: *NCMC* new corporative medical care, *CEMI* city employee medical insurance, *CRMI* city resident medical insurance, *URMI* urban and rural medical insurance, the power for the differences in the percentages of inpatient cancer care expenditure reaching given cutoff percentages of family income among different groups was calculated using *χ* ^2^ test

### Multivariate regression analysis of ICC expenditures

Table 5 provides multivariate regression statistics of ICC expenditures. Level of hospital and household income showed independent associations with direct, indirect and total ICC expenditures; while education, with direct and total expenditures; and region, with indirect expenditure; and reimbursement ratio, with indirect and total expenditures.

**Table 5:** Multivariate regression analysis of ICC expenditures

Factors	Direct expenditure			Indirect expenditure			Total expenditure		
	B	t	P	B	t	P	B	t	P
Constant	1.611	8.404	<0.001	0.805	2.386	0.018	1.630	7.688	<0.001
Age	0.000	0.458	0.648	0.002	1.680	0.094	0.000	0.658	0.511
Gender	−0.025	−1.664	0.097	0.032	1.278	0.202	−0.010	−0.607	0.544
Region	0.006	0.441	0.659	0.064	2.631	0.009	0.011	0.708	0.479
Education	−0.021	−2.715	0.007	0.005	0.399	0.690	−0.018	−2.090	0.037
Reimbursement ratio	0.000	1.128	0.260	−0.002	−3.155	0.002	−0.002	−6.507	<0.001
Ln (household income)	0.233	2.882	0.004	0.356	2.514	0.012	0.236	2.643	0.009
Level of hospital	0.068	7.768	<0.001	0.080	5.284	<0.001	0.066	6.865	<0.001

## Discussion

This study revealed useful information about ICC in Anhui, a typical inland province of China. Firstly, the study highlighted the magnitude of ICC burden on the affected, related and the whole society and that cancers characterize low incidence, moderate service use and high expenses. Published incidence rate of all cancers has been less than 300 in every 100 thousand population [25]; while our findings of a total of 318 person-times of ICC out from 60,678 residents within 1 year time translates into a hospital admission rate as 524.08 per 100 thousand population, almost twice that of total cancer incidence rate. Similarly, although total ICC episodes took up only 6.33 % of all inpatient service episodes in our study, direct ICC expenditure accounted for 10365.14 RMB (or $1672) per person annually and 13.30 % of all inpatient care expenditures for the whole population; and per case expenditures on ICC turned out to be 2.63 times that on other diseases. This unique nature of cancers has a couple of implications. On the one hand, the relatively low incidence of the epidemic combined with a general lack of researches on the service use and costs makes it easy to under-estimated the overall burden and hence importance of ICC burden. On the other, the high per case costs may imply opportunities for service savings as well as quality control. Although our estimated per case costs ($1672) on cancer falls a little bit higher than the bottom line of the range of international estimations ($1643 to $7705) in absolute monetary terms, the proportion of ICC expenditure in total health expenditure (13.30 %) from our study is much higher than that from other nations (e.g., 5 % in the USA) [26]. This may have profound impact on both the patients and the health systems in China.

Secondly, the current study documented substantial differences in person-times and person-days of ICC between different sub-groups. These two indicators showed a clear increasing trend from younger to older age groups. This is consistent with epidemiological findings that most cancer incidence rates increase exponentially with age [25]. And co-morbidities may also have played some roles in the cost differences across the age groups since the older the age the greater the chances of the cancer patients to have co-morbidities. This finding leads to an important inference that as China’s population ages, ICC burden will grow rapidly. For gender subgroups, this age-related trend applied only to males but females. This may be explained mainly by the difference in types of cancers among males and females. Breast and cervical cancers accounted for 30.71 % of all the cancers among women. These two types of cancers are linked, to a large extent via direct or indirect paths, with reproductive hormones (e.g., estrogens) that start to decrease after some age around 50 [27]. Compared with age and gender discrepancies, that may be attributed mainly to differences in incidence rates, huge variations in person-times and days of ICC among residents covered by different insurance schemes merit particular attention since these differences may due largely to service utilization rather than physical and pathogenic factors. In China, it is commonly believed that beneficiaries of the medical insurance for city employees and residents enjoy the highest reimbursement ratio and the loosest process for claiming reimbursement; followed by the new corporative medical care scheme and others. This order is consistent with that of per thousand capita ICC person-times and days indicating that medical insurance systems may have played an important role in shaping ICC utilization. Although the differences between subgroups of household income and education were statistically significant, they did not exhibit clear trend. This may because income and education exerted mixed effects on ICC use since higher income and education may mean not only better ability obtaining ICC but also lower chances to develop cancer(s) [28]. With regard to greater admission rates and length of stay (days) for urban verses rural residents, possible explanations may include higher total cancer incidence rate, easier access to ICC, and better household income, education and insurance etc. in urban than rural areas.

Thirdly, the study revealed noteworthy characteristics in per case ICC expenditure. The variations in expenditures on ICC provided by different level of hospitals turned out to be the greatest among all the subgroups studied, being 2142 RMB for ICC at township hospitals compared with 17,133 RMB for ICC at province level hospitals. This may be explained mainly by: a) lower level hospitals charged their patient at much cheaper price [29]; b) patients with more serious conditions tended to seek ICC from higher level hospitals [30]. Given such huge gaps, there is a clear need for guidelines or regulations directing cancer patients to appropriate level of hospitals. However, China lacks relevant guidance or regulations and selection of hospitals depends primarily on individual physicians’ experiences or patients’ understandings. Per case expenditure on ICC also showed statistically significant variations among patients with different household income and type of medical insurance systems. Patients with higher household income spend more on a single episode of ICC may because they faced looser financial constraint in choosing diagnosis and treatment procedures. The reason why patients belonging to enrollees of the new corporative medical care and the city resident medical insurance incurred relatively lower per case direct expenditure may because mainly that these two systems had enacted the strictest policies and audit procedures in refunding medical care expenses [31]. Although the diagnosis and treatment procedures and regimens vary from cancer to cancer, yet the differences in per case direct expenditure across cancer types turned out to be statistically non-significant. This contradicts similar findings derived from other countries [32] and merits further researches. It may hint a peculiar phenomenon of ICC in which treatment decisions were not based primarily on the need of the patient under concern but on the actual or perceived willingness to pay for his or her treatment.

Fourthly, the study portrayed catastrophic nature of ICC expenses (Table 4). Expenditures on ICC accounted for 13.30 % of that on all inpatient care and per-case expenses on ICC was 2.63 times that on all other diseases. After excluding expenditures refundable from various insurance systems, as high as 20.6 % of the out-of-pocket payment due to ICC turned out to be catastrophic to the patients according to the World Health Organization standard (40 % of total household income) [24]. This included only direct and indirect cost of ICC without taking into account expenditures on outpatient care, self-care, repeated hospital admissions and others. The burden of ICC was most devastating for patients who were: enrollees of other and URMI (urban and rural medical insurance) medical insurance systems; 65 to 74 years old and 45 to 54 age group; and prefecture or higher level hospital patients. Combined with the common belief that there lack radical cures for cancers, this catastrophic nature of ICC expenditures may make the affected and related feel most hopeless and helpless [33] and this feeling has profound implications for clinicians as well as policy-makers.

This study has both strengths and weaknesses. Perhaps, the biggest advantage of the current study relates to its “household-based” data collection. Most published papers addressing inpatient care expenditures belonged to “hospital-based” investigations [4, 9, 10, 12] and hence suffered from major selection biases since ICC expenditures vary greatly across hospitals and drawing a sample of ICC cases from hospitals in proportion to ICC cases actually happened in the community is almost impossible. Although estimating ICC burden via “household surveys” avoids the sampling difficulties faced with “hospital-based” studies, it is prone to recall biases. It is very easy for some respondents to forget part of the expenses even whole ICC episodes happened during a whole year. It is also quite often in China that, for fearing of potential worries or distress, cancer diagnosis are not disclosed to part of the household members (especially the patients themselves). These may have resulted in under estimations of the ICC costs. Future studies in this regard should take appropriate measures in minimizing this recall bias, e.g., selection of the most educated or capable household members as the respondents, use of carefully designed categories of expenditures as reminders, and triangulation. Another drawback of the study concerns the size of subjects studied. Although the survey covered 60,678 residents, it identified only 318 ICC episodes which included very limited cases of rear cancers, e.g., nine cases of liver cancer. So, readers are well cautioned about the potential statistical variations interpreting our estimations especially expenditures or length of stay for subgroups.

## Conclusion

Cancers characterize low incidence, moderate service use and high expenses. There exist substantial differences between subgroups and part of these variations cannot be explained by pathological factors. There are indications that treatment decisions on ICC were not based primarily on the need of the patient under concern but on the actual or perceived willingness to pay. ICC expenses are catastrophic in nature to a large part of patients. There is a clear need for more effectively regulating cancer-related medical practices and service seeking behaviors.

### Availability of data and materials

The raw dataset of all the inpatient cancer care cases is accessible from Additional file 1.

## Appendix 1

**Table 6:** Per-case inpatient care expenditures by types of cancers

Type of cancer	Cases	Direct expenditure			Indirect expenditure		
		LNmean	LNSD	Mean (RMB)	LNmean	LNSD	Mean (RMB)
Lung cancer	50	2.20	0.14	8505.77	1.87	0.25	651.96
Stomach cancer	47	2.26	0.12	14627.70	1.96	0.16	1234.37
Colorectal cancer	41	2.23	0.13	10584.85	1.86	0.21	616.44
Esophageal cancer	27	2.22	0.13	10317.53	1.88	0.20	714.95
Cervical cancer	24	2.25	0.11	12741.51	1.99	0.11	1520.87
Other cancer	129	2.21	0.12	9482.52	1.94	0.18	1054.51
*P* values		0.208			0.014		
All Cancers	318	2.224	0.124	10365.136	1.922	0.194	929.863

Note: *LNmean* the mean of Ln(Ln(x)), where x stands for individual health expenditures, RMB stands for Chinese yuan, the power for the differences in per-case inpatient care expenditures among different types was calculated using ANOVA

## Appendix 2

**Table 7:** Per-case inpatient cancer care expenditures by different groups of patients

Grouping criteria	Direct expenditure			Indirect expenditure		
	LNmean	LNSD	Mean (RMB)	LNmean	LNSD	Mean (RMB)
Age (years)						
Under 45	2.23	0.12	10743.79	1.95	0.20	1162.20
45 to 54	2.24	0.13	12225.86	2.02	0.16	1843.87
55 to 64	2.20	0.12	8423.02	1.85	0.20	578.89
65 to 74	2.26	0.12	14231.26	1.93	0.19	975.59
75 or over	2.21	0.11	9126.47	1.93	0.14	957.97
*P* value	0.075			<0.001		
Type of health insurance						
NCMC	2.21	0.12	8907.97	1.91	0.20	872.20
CEMI	2.26	0.11	14846.80	1.93	0.20	998.14
CRMI	2.20	0.11	8096.26	1.88	0.12	681.10
URMI	2.25	0.17	13183.24	2.02	0.19	1943.92
Others	2.31	0.15	23120.53	2.00	0.26	1577.44
*P* value	0.001			0.043		
Household income (quartile)						
Q1	2.18	0.13	7200.36	1.86	0.24	636.12
Q2	2.23	0.11	11243.20	1.95	0.17	1104.75
Q3	2.23	0.12	10980.39	1.91	0.17	852.85
Q4	2.25	0.13	12977.18	1.96	0.19	1243.66
*P* value	0.009			0.051		
Level of hospital						
Township	2.04	0.13	2142.30	1.66	0.43	193.00
County	2.19	0.11	7700.41	1.86	0.17	613.14
Prefecture	2.25	0.12	12974.11	1.98	0.16	1379.28
Provence	2.28	0.11	17132.95	1.98	0.18	1361.48
*P* value	<0.001			<0.001		
Education						
No education	2.24	0.12	12125.86	1.93	0.18	993.32
Primary school	2.21	0.13	8822.87	1.90	0.21	804.18
Middle school	2.23	0.12	11409.51	1.93	0.18	975.92
Secondary or higher school	2.22	0.12	10042.12	1.94	0.18	1070.38
*P* value	0.245			0.551		
Total	2.22	0.12	10365.14	1.92	0.19	929.86

Note: *NCMC* new corporative medical care, *CEMI* city employee medical insurance, *CRMI* city resident medical insurance, *URMI* urban and rural. RMB stands for Chinese yuan; the power for the differences per-case inpatient cancer care expenditures among different groups was calculated using ANOVA

## Additional file

Additional file 1: Raw dataset of inpatient cancer care costs and related variables studied. (XLSX 32 kb)

## Abbreviation

ICC: inpatient cancer care
FBC: financial burden of cancers
SD: standard deviations
URMI: urban and rural medical insurance
